# Imparting carrier status results detected by universal newborn screening for sickle cell and cystic fibrosis in England: a qualitative study of current practice and policy challenges

**DOI:** 10.1186/1472-6963-7-203

**Published:** 2007-12-13

**Authors:** Hilda Parker, Nadeem Qureshi, Fiona Ulph, Joe Kai

**Affiliations:** 1 Division of Primary Care, University of Nottingham, Graduate Medical School, Derby City General Hospital, Uttoxeter Road, Derby, DE22 3DT, UK

## Abstract

**Background:**

Universal newborn screening for early detection of children affected by sickle cell disorders and cystic fibrosis is currently being implemented across England. Parents of infants identified as carriers of these disorders must also be informed of their baby's result. However there is a lack of evidence for most effective practice internationally when doing so. This study describes current or proposed models for imparting this information in practice and explores associated challenges for policy.

**Methods:**

Thematic analysis of semi-structured interviews with Child Health Coordinators from all English Health Regions.

**Results:**

Diverse methods for imparting carrier results, both within and between regions, and within and between conditions, were being implemented or planned. Models ranged from result by letter to in-person communication during a home visit. Non-specialists were considered the best placed professionals to give results and a similar approach for both conditions was emphasised. While national guidance has influenced choice of models, other factors contributed such as existing service structures and lack of funding. Challenges included uncertainty about guidance specifying face to face notification; how best to balance allaying parental anxiety by using familiar non-specialist health professionals with concerns about practitioner competence; and extent of information parents should be given. Inadequate consideration of resource and service workload was seen as the main policy obstacle. Clarification of existing guidance; more specific protocols to ensure consistent countrywide practice; integration of the two programmes; and 'normalising' carrier status were suggested as improvements.

**Conclusion:**

Differing models for communicating carrier results raise concerns about equity and clinical governance. However, this variation provides opportunity for evaluation. Timely and more detailed guidance on protocols with clarification of existing recommendations is needed.

## Background

Part of the newborn bloodspot programme [1], universal newborn screening for sickle cell disorders (SCD) is now fully implemented across England [2] and will be for cystic fibrosis (CF) [3] by mid-2007. In addition, a linked antenatal haemoglobin disorder (HD) screening programme is being rolled out; universal in areas with a high prevalence and selective in low prevalence areas. High prevalence areas are those where sickle cell disease is estimated to affect more than 1.5 per 10,000 pregnancies and low prevalence those with less than 1.5 per 10,000. Newborn screening aims to identify babies affected by these conditions; however screening also identify infants who are carriers. While concerns have been raised about identifying carriers of genetic conditions by population screening and how to (even whether to) communicate results [4-7], English policy is that parents have to be informed. Despite decades of universal newborn screening for SCD in the USA, and more recently for CF, there is no clearly established model for effective communication of carrier status internationally [8].

Both SCD and CF are recessively-inherited disorders and carriers are healthy. Newborn screening for SCD identifies all carriers of structural haemoglobin variants (screening identifies carriers of 'unusual haemoglobins' of which the most common is Sickle Cell; for ease of reference, SCD is used in this paper when referring to all unusual haemoglobins detected by screening) but not thalassaemia carriers and there is no available method of testing without detecting carriers. The national protocol for CF screening in England aims to identify a maximum number of children with CF whilst minimising the number of carriers. The protocol involves an initial immuno-reactive trypsinogen (IRT) measurement which identifies babies at high risk for CF. These samples are further tested by a two-stage DNA screen for a small panel of CF mutations. Those with two mutations will have CF. For those with only one mutation or no mutation detected but with a very high initial IRT, a second blood sample will be requested and a further IRT measurement performed at 21–28 days when it is more discriminatory. While an elevated IRT itself does not select in favour of carrier status, most of these babies will be carriers defined by limited DNA testing. However, because not all mutations are identified, a small proportion of those defined as carriers may actually have two CF variants and have the condition (because they have inherited two CF variants, one identified by IRT and one not identified).

With a SCD incidence of 1:2400 affected babies per year in the UK, the NHS Sickle Cell and Thalassaemia Screening Programme estimate that about 8000 newborn carriers were detected in 2006 from about 550,000 babies screened [9]. Cystic fibrosis has a slightly lower incidence (1:2500 affected babies per year born in the UK) with approximately 1 in 25 of the population estimated to be a carrier of which only a small proportion will be identified by newborn screening [3]. In England annually about 240 babies with CF are born [10] and current screening protocols are expected to identify an equivalent or slightly higher number of carriers. Thus, compared to CF, numbers of newborn SCD carriers detected are considerable. For example at a regional level, during a 12-month period 573 SCD carriers and 16 CF carriers were detected in a population with an annual birth rate of 70,000 [11].

Prior to national programmes, newborn screening was mostly offered on an ad hoc basis, with universal CF screening available to 20% of babies (areas served by laboratories in East Anglia, East Midlands, South Yorkshire and Leeds) for over 15 years and over 10 years for SCD in some areas in London, East of England and Birmingham. Over this period, although practice for informing parents of their infants' carrier status has varied according to condition and locality [12], the need for clear protocols for communicating carrier information is recognised [13]. In England, thus far, guidance issued by the UK Newborn Screening Programme Centre recommends that carrier results should be given to parents 'as soon as possible; by a well-informed health professional; in person, or by phone, and followed up in person as soon as possible; and supported by written information' [10]. This guidance does not distinguish between CF and SCD carrier status. However there are no data on models being used, extent of variation, and experience of implementation following the advent of universal screening across England.

This paper reports findings from a descriptive study, part of a larger study funded by the Health Technology Assessment programme [14] also exploring parents' and health professionals' experience, on models for giving newborn carrier results and emerging policy in this context.

## Methods

Semi-structured telephone interviews were conducted (by HP) with the Regional Child Health Co-ordinator from each of the nine English health regions during the second half of 2006. Consent to be interviewed was initially obtained by email and again verbally on tape at the start of the interview.

Participants were invited to reflect on the extent of regional implementation of CF and SCD newborn screening, actual or proposed models for giving results, the need for condition specific models, who should give the results, and suggestions for improving current practice and policy. Respondents were also able to raise other issues of importance relevant to the subject. Where informants were unable to provide sufficient details, brief telephone calls or emails to specialist services were used to acquire supplementary information. Interviews were tape-recorded and transcribed verbatim. Data were thematically coded and analysed according to emergent themes. Interviewees were invited to give feedback on a draft version of this paper prior to submission for publication.

The study was approved by the West Midlands Research Ethics Committee.

## Results

### Description of regional models

Participants reported a variety of models, proposed or already operational, for imparting carrier results (see table 1 for summary of practice across England). Of note is diversity of models both within and between regions and both within and between conditions. Only one region is implementing the same model for both conditions. All regions are informing parents of CF carriers in person compared to a range of methods, from notification by letter alone to personal contact, for SCD carriers. Health Visitors (HVs) have a prominent role, including family's usual HV and those specially trained to communicate carrier results.

**Table 1:** Proposed English Regional methods for imparting newborn carrier results

**Region**	**SCD carriers**	**Sceening started in**	**CF carriers**	**Screening started in**
North West	**In person **by purpose trained health visitor	2005	**In person **by purpose trained health visitor	Not yet started at time of interview
West Midlands	**In person **by Haemoglobinopathy counsellor **OR** **Letter **with result plus appointment for counselling	2004	**In person **specialist counsellor plus family health visitor	Not yet started at time of interview
South West	**In person **by family health visitor or Haemoglobinopathy counsellor **OR** **Letter **with result plus option to attend Haemoglobinopathy service	2004 – 2006	**In person **by genetic counsellor or antenatal screening co-ordinator	Not yet started at time of interview
London	**In person **by family health visitor or Haemoglobinopathy counsellor **OR** **Letter **with result plus appointment for counselling, or option to attend specialist service, or 'drop-in' clinic	1995 – 2005	Undecided at the time of data collection	Not yet started at time of interview
North East	**In person **by Haemoglobinopathy counsellor plus family health visitor	2005	**In person **by purpose trained health visitor plus family health visitor	Not yet started at time of interview
East of England	**In person **by Haemoglobinopathy counsellor or purpose trained health visitor **OR** **Letter **with result plus appointment for counselling	1997 – 2005	**In person **by specialist CF nurse	1980's in Cambridge-shire
South Eastern	Haemoglobinopathy counsellors contact and inform parents (details on exact methods not collected)	2003 – 2006	**In person **by purpose trained midwife	2006 in Thames Valley
East Midlands	**In person **by General Practitioner plus referral to clinical genetics or follow-up by Haemoglobinopathy counsellor	2004	**In person **by screening specialist nurse	1989
Yorkshire and Humber	**In person **by purpose trained health visitor or Haemoglobinopathy counsellor **OR** **Letter **with result plus appointment for counselling	2004	**In person **by family health visitor plus specialist CF nurse or by family health visitor alone	1997 – 2007 1989 (South Yorkshire)

### Factors shaping choice of method

#### National guidance

National guidance (as issued by the UK Newborn Screening Programme Centre [10]) for implementation most commonly influenced choices of methods for communicating carrier results. The perception was that guidance was more specific for the CF compared to the SCD programme. However, experience of implementing SCD screening first has informed thinking about CF.

*'... because there were lines being drawn in the sand as to who should actually do this information, who should actually give this information and obviously we knew that Cystic Fibrosis was coming...so I adopted the model for the Sickle screening programme as well.' *(CHC 04)

#### Resource constraints

Inadequate funding has affected implementation of both the delivery of screening and in particular the choice of methods for communicating carrier results. This has necessitated efforts to secure funding from local sources; often short term and dependent on an individual manager's resourcefulness or sway within the local health system. Where no additional funds could be realised, existing staff have had to take on communication of results in addition to their usual workload. Inevitably, these financial constraints have, in some regions, led to compromise and 'quick fix' models for communicating carrier results.

*'...I don't think there was due consideration given to the workload associated with giving carrier results and I think that was an oversight. There doesn't seem to have been any thoughts on how it would be... It needs to be properly accounted for, like we introduce services and they give 4 quid a baby for the lab but it affects every different component part of the service. It affects the midwives and their counselling, it affects the health visitors giving the results and it affects the child health record departments who have to adapt their systems of working to record the results.' *(CHC 02)

'... *it's *[informing carriers]*all done on goodwill, the PCTs are asking where's the funding for this? And obviously it does take up some time, some practitioners' time.' *(CHC 04)

#### Low or high prevalence areas for Haemoglobin Disorders

Specialist services have been operational in high prevalence HD areas long before the introduction of universal newborn screening. Bringing distinct advantages, such as expertise and referral protocols, a consequence is that regional plans for models of carrier results have to incorporate existing practice and organisational structures, resulting in less scope for innovation in some areas. Requesting changes to existing practice was a challenge, leading some interviewees to prefer starting service planning from scratch.

'...*it was easier to do the area that was a blank sheet because then you could do how best fitted what the geography and, you know, where the funds and all those sorts of things available were and you've also got some handle on what they do and can say what they should or shouldn't do. Whereas when there's already something in place it's harder isn't it?' *(CHC 08)

In contrast, there were concerns about reporting results in low prevalence HD areas due to lack of resources and practitioner knowledge. Thus, although low prevalence settings provided opportunities for trying out new models of result giving, in some areas urgency of need necessitated rapid implementation before localities were sufficiently prepared to deliver results.

*'Our real problem has been our low prevalence areas...it was little bit hit and miss to be quite honest. We had a case... where we found 60 children hadn't been given results. And that was a bit...because people didn't know quite what to do with it, how to do it...' *(CHC 05)

#### Local consultation and preferences

Regional implementation groups were a common mechanism for discussion and planning of proposed models. For some, the challenge was to find a fit between national guidance and local resources and preferences. Regions who consulted widely about this specific issue and ensured extensive health professional (e.g. Directors of Public Health, Primary Care Trust screening leads, paediatricians, heads of midwifery and health visiting, etc) engagement found that the process benefited implementation of the screening programme as a whole.

*'I think without doubt the implementation has brought more people around the table...and trying to ensure that there is linkage and involvement across the whole of the screening profession. So making sure that every professional group, primary, tertiary and secondary level specialists have been involved in that decision making has been beneficial.' *(CHC 07)

### A role for non-specialists

With some exceptions, most interviewees expressed a strong preference that conveying carrier results should be a task undertaken by non-specialist health professionals. During interviews respondents used the term 'specialists' when referring to genetic counsellors, Haemoglobinopathy counsellors and Cystic Fibrosis nurses and regarded all other health professionals involved in communicating carrier status information as 'non-specialists'. Key to this position was the view that carriers were healthy. Utilising a specialist practitioner in this role could cause parents to believe that their baby was ill and increase their anxiety. Specialist time was more appropriate for providing further information to families who wanted to know more or wanted to discuss future reproductive decisions. While support for non-specialists giving results was consistent, informants were uncertain about how best to balance allaying parental anxiety by using a non-specialist and concerns about practitioner competence.

'*And we would like for them *[specialist HD counsellors] *to spend more time doing the specialist stuff that a health visitor couldn't possibly do... for the carriers it's quite a large workload and yet it doesn't need super-specialist people, it needs somebody with some extra training and some expertise and it's sort of half-way house.' *(CHC 06)

Respondents working in regions where specialists were currently involved in giving results did not see this as a problem, though they were not insistent that specialists should be involved. In one region concern had arisen about non-specialists giving CF carrier results because of the small risk that some carriers may be affected.

The importance of involving the family's usual HV in giving results was highlighted as the best way to minimise parental anxiety; either as the sole professional giving the results, or visiting the family together with a specialist or purpose trained non-specialist. Ensuring appropriate training for HVs was an important consideration. Whether to train all to give results, knowing that some may never come across a carrier case, or to concentrate training to a selected group who would take on this role and accompany the family HV remained an ongoing debate for some regions.

'...*it seems to me that the best person to give the results so that it isn't worrying is in the middle of a routine health visitor visit without the phone call to say, ' hey can I see you', especially because in a sense that's making anxieties. But how do we maintain competence if even at local level you know no health visitor is going to be doing it every week say or even once a month so I think there's actually a real dilemma... ' *(CHC 06)

### Condition specific or same approach for both conditions

None of the interviewees were in favour of separate models for the two conditions. Two respondents, who had not yet implemented CF newborn screening, wanted to await further experience while others expressed strong preference for similar models and for close working between the two screening programmes. Another suggested that the difference between carrier results for the two conditions was over stated.

*'...I think there should be *[the same model] ...*I've thought about this quite a lot because with cystic fibrosis the results can be difficult to interpret and some of the mutations the significance of those isn't known. But then I thought with sickle cell screening some of the haemoglobin variants, the significance of those is unclear so the results of that can be equally as difficult to interpret and not always straightforward'*. (CHC 01)

A common view was that there were more similarities, such as carrier status, recessive inheritance, and skills required to inform parents, than differences between the conditions. Therefore, it appeared logical to have the same protocol and organisational structure, albeit with some variation, for giving results.

*'Well when we were putting the whole system *[SCD newborn screening]*into place in the back of our mind all the time was the fact that CF has got to roll out and it makes sense to use the same mechanism because the counselling skill is the same isn't it? You know, telling somebody that there's a problem with their baby and this is the genetics and you know, that sort of skill...a counselling skill is a counselling skill really isn't it?' *(CHC 08)

*'I would think it should be the same method, you know, I think it should be. Ideally I mean it's the same recessive condition that you're describing, the same genetics involved so you know I'd be of the opinion you could do both.' *(CHC 03)

Parity in methods for the two conditions was also seen as a way of addressing longstanding inequity in NHS service provision for HD compared to CF.

'*I think it *[methods for giving carrier results]*should be standard but...because I do find it...I do find it personally irritating that there's this difference between sickle cell and CF. And professionally I think, well sickle cell is a genetic condition so why don't clinical genetics see it as their remit a little bit more because it is an inherited condition... but that's always been the way. Sickle cell services seem to have existed running parallel to clinical genetics and erm...so as I say, it's *[Haemoglobinopathy Disorders]*probably a bit of Cinderella area...' *(CHC 01)

### Suggestions for advancing practice and policy

The need to clarify what was meant in the guidance by 'communicating in person' was a priority. Personal informing was seen as a costly process and some respondents suggested that an appropriate leaflet with contact details for further information could be as effective as a personal visit. Others considered using only written information unsatisfactory as varying reading levels would increase misunderstanding and service providers would not know that parents had received the information.

*'...I think we need a better definition of what is 'communicated in person' because you could interpret that, couldn't you, as here's the leaflet read it, it could be here's the leaflet shall we go through it together...erm...through the whole thing about you know a specialist ringing up and saying nothing to worry about but I need to see you...erm... The other thing is in the 'in person', I mean if you've got a family that don't speak English...if the health visitor doesn't speak their language but goes in with a leaflet in the right language you know, is that a face to face contact or whatever?' *(CHC 06)

A nationally agreed protocol for informing carriers with clear expectations of what information needs to be communicated to parents and practitioner roles, more detailed than current guidance and similar for both conditions, was suggested.

'*Well, it's about clear expectations. About making sure that there is clear linkage of what you do next. I think if you are going into a family to give a result, it is not just good enough to give a result and to give a leaflet. You must provide the next level of intervention. And that next level in intervention is about listening, and then signposting and very clearly where you go next. And it isn't just about saying 'go to your GP and they might refer you to clinical genetics' because some GPs may not. So I think that needs to be really erm...agreed before we sort this out. What is the kind of things if people want further support? The other thing that needs to be clear, so that the Health Visitors are very clear, is about we are not asking them to become genetics experts and we are not asking them to become Sickle Cell experts. ... we need to have some very clear role boundaries of what is expected and that is agreed boundaries and part of it is you may have a Health Visitor that is really interested and wants to do a lot more but is it appropriate?*' (CHC 05)

Explicit national policy regarding cascade screening (testing of other family members), and whether and how to report results detecting non-significant haemoglobin variants would also be helpful. More practical proposals included scripts on what parents should be told, especially when handling contentious scenarios such as non-paternity; a leaflet for parents of SCD carriers (similar to the current CF leaflet); simplification of current leaflets; and review of when (timing) parents are told what the bloodspot test is for.

As part of the call for continuity across both conditions, integration of the two programmes was presented as imperative to ensure consistency in practice (and arguably, equity).

*'The other stuff that we really, really need to do is to not to do something totally different for CF and for Sickle because we do an awful lot of going down different pathways and I don't think that makes any sense...the integration of the two programmes is just so important because it's really hopeless if they don't erm...because you get these mixed messages and you know you get it all being very special and very different. I mean it's one of the issues I think all children with chronic disease or carriers, they've got more in common through being children than they have in having a disease erm...and it seems to me we shouldn't be taking people into different pathways simply because they've got one type of disease.' *(CHC 06)

Respondents felt that giving results should be 'normalised' and incorporated into usual health care practice. Where possible, lessons could be learnt from other screening programmes where results may be equally worrying. Increased public awareness of the conditions and screening programmes was also mentioned as a way of allaying parental concerns about carrier status.

*'I think the biggest problem for the counsellors is getting across to the parents that carrier status isn't a disease and I think if we could raise the public's understanding of what Sickle Cell was, I think that would help them enormously because *[otherwise]*they've got to start from zero haven't they really. And bring parents up because it can come out of the blue can't it, they don't even know anything about it and then you're telling them that there's something wrong with their perfect baby.' *(CHC 08)

Although confident that this task was within a non-specialist remit, participants noted concerns about practitioner competence. In particular, General Practitioners' (family physicians) limited knowledge about the implications of carrier status was perceived as concerning and needed to be addressed.

Creation of a new post for a designated health professional within a specific locality, described as a 'newborn bloodspot practitioner', to take responsibility for newborn screening carrier results for all conditions was proposed. This was seen as a practical way forward to facilitate continuity in liaison with laboratories and other stakeholders, and maintenance of professional competence in result giving.

The pressing need for research evidence to inform current practice was emphasised. Participants wanted to know the cost-effectiveness of various models and parental preference for delivery format and information content.

## Discussion

Recent US reports on CF newborn screening conclude that policymakers must consider the need for genetic counselling services and ensure adequate resources to support information giving prior to introducing newborn screening programmes [15,16]. European experience echoes this call for clearly defined referral pathways to support maximum gain and minimise negative outcomes [17].

Research with parents following newborn screening supports disclosure of carrier status [18] and preference for being informed by a familiar, non-specialist, health professional [19]. Prior warning to expect the result [8] and the need for concise information about newborn screening to be given during the antenatal period [20,21] are further parental needs. In a retrospective study of parental attitudes following carrier identification, no long-term adverse outcomes were noted in the majority of families [22]. However, the cost-effectiveness of face to face consultation has been questioned [23] and rigorous evidence on methods for communicating carrier results remains lacking.

Our data on proposed models for imparting newborn carrier results across England suggest marked diversity, both within and between regions, and within and between conditions. Although influenced by national policy, other factors have shaped practice leading to pragmatic rather than ideal choices. Despite overwhelming support for a similar process for both conditions, only one region was implementing a similar model for both conditions and variance of methods was particularly noticeable for SCD carriers. This creates concerns about equitable service provision.

Policy and accompanying funding may not address the full implications of introducing new newborn screening programmes in relation to giving carrier results and dealing with parental information needs. As funding has supported only the front end costs of the process, such as laboratory tests, health communities have had to find additional resources within existing budgets, which may result in less than ideal models for giving results.

Though modest in scale, these interviews with stakeholders tasked with coordinating the implementation of newborn screening programmes are likely to have covered the full range of current models for giving carrier results in England. Our respondents' views may not have embraced perspectives beyond their own roles, for example those from antenatal screening or primary care, though, this would be unlikely to change the key messages and issues for debate raised here.

## Conclusion

Universal newborn screening for SCD and CF heralds a new era for genetic screening in England [24]. While early diagnosis of children affected by these disorders is the aim, screening of populations will increasingly require provision of information for those identified as gene carriers (and their families) with consequent challenges for appropriate service models and workforce planning and training.

Findings from this study raise important policy and practice issues for professionals with strategic and operational responsibility for implementing newborn screening. For example, the costly process of reporting in person, especially in terms of numbers of SCD carriers; challenges of maintaining non-specialist informant competence given small number of carriers per condition per year (while carrier numbers will be far greater for SCD relative to CF, when translated to a primary care practice level, carriers of either condition will still be a rare occurrence within a GP or HV caseload); the need for more detailed national protocols; and equity and clinical governance concerns.

Our description of current practice may encourage shared learning as newborn screening is rolled out, as well as a context to stimulate evaluation and research about methods for imparting carrier results. Immediate priorities are for practical support for implementation such as good information leaflets, national protocols for information giving, and additional resources to support carrier result communication. Evidence for best practice when imparting results will emerge over time as practice models evolve and adjust.

## Competing interests

The author(s) declare that they have no competing interests.

## Authors' contributions

HP devised the methodology, conducted and analysed the interviews and lead the writing, JK conceptualised the idea as principal investigator for the research of which this study forms part and assisted with drafting the paper, NQ contributed to design and assisted with writing, FU helped with analysis and writing. All authors read and approved the final manuscript.

## Pre-publication history

The pre-publication history for this paper can be accessed here:


